# Sleep Spindles Characteristics in Insomnia Sufferers and Their Relationship with Sleep Misperception

**DOI:** 10.1155/2016/6413473

**Published:** 2016-07-11

**Authors:** Marie-Pier Normand, Patrick St-Hilaire, Célyne H. Bastien

**Affiliations:** ^1^École de Psychologie, Université Laval, Québec, QC, Canada G1V 0A6; ^2^Laboratoire de Neurosciences Comportementales Humaines du Centre de Recherche de l'Institut Universitaire en Santé Mentale de Québec, Québec, QC, Canada G1J 2G3

## Abstract

Cortical hyperarousal is higher in insomnia sufferers (INS) than in good sleepers (GS) and could be related to an alteration in sleep protection mechanisms, like reduced density or altered characteristics in sleep spindles. The deficient sleep protection mechanisms might in turn enhance underestimation of sleep. This study's objective was to document sleep spindles characteristics in INS compared with GS and to investigate their potential role in sleep consolidation and misperception. Seventeen individuals with paradoxical insomnia (PARA-I), 24 individuals with psychophysiological insomnia (PSY-I), and 29 GS completed four consecutive polysomnographic nights in laboratory. Sleep spindles were detected automatically during stage 2 and SWS (3-4) on night 3. Number, density, duration, frequency, and amplitude of sleep spindles were calculated. A misperception index was used to determine the degree of discrepancy between subjective and objective total sleep times. Kruskal-Wallis *H* tests and post hoc tests revealed that PARA-I had significantly shorter sleep spindles than GS but that PSY-I and GS did not differ on spindles length. A standard multiple regression model revealed that neither sleep spindles characteristics nor objective sleep measures were predictive of sleep misperception. A longer duration of spindles could reflect a higher gating process but this hypothesis still needs to be confirmed in replication studies.

## 1. Introduction

Insomnia is the most prevailing sleep disorder. Approximately 30 to 48% of adults complain of insomnia symptoms [1] and 10% have symptoms significant enough to meet the diagnostic criteria of insomnia syndrome [2]. Insomnia is characterized by an inability either to fall asleep and/or to maintain sleep throughout the night and/or a waking time earlier than desired. The complaint in the quality or quantity of sleep must be present for at least 3 nights per week during the last 3 months and associated with daytime impairments [3]. Although polysomnography (PSG) is considered the gold standard for the objective and quantitative assessment of sleep, some of its aspects remain understudied in the insomnia population. In fact, even if insomnia sufferers (INS) spend more time in stage 1 and less time in stages 3 and 4 and display more frequent changes in sleep stages [4–6], sleep protection mechanisms and their role towards the altered sleep of INS have been scarcely studied. Strangely, these sleep protection mechanisms might be deficient in INS, impairing sleep soundness. Less sound sleep could then be linked to one's ability to adequately perceive sleep.

Sleep protection mechanisms can be measured using phasic events such as sleep spindles, which are nonrapid eye movement (N-REM) electroencephalographic oscillations appearing only during sleep. Although they can be observed in stages 2, 3, and 4, they are hallmarks of stage 2 [7]. They are distinctively detected from the electroencephalogram (EEG) background as waxing and waning transient events from 11 to 15 Hz and lasting between 0.5 and 2 seconds. Their presence in the EEG reveals the wake-sleep transition as neural synchronization becomes higher [7, 8]. Sleep spindles have usually been attributed two functional roles. Some researchers suggest an important role in learning and plasticity (for a review, see [9]). On the other hand, others postulate a role in sleep protection by the inhibition of sensory processing, more specifically the disengagement of disrupting and/or intrusive stimuli [10–12], which has been supported by some empirical studies [13, 14]. In INS, the number and density of sleep spindles have been reported as being similar to those of GS [15], suggesting that this sleep protection mechanism is intact in INS.

However, characteristics other than number and density (amplitude, frequency, and duration) are less often examined. In fact, information about the respective role of these characteristics is still sparse and limited. For instance, the probability of nRT neurons to be involved in the first cycle of a sleep spindle is negatively associated with the spindle duration [16]. Also, frequency is related to the rate of hyperpolarization: that is, the longer the hyperpolarization is, the slower the spindle frequency will be [17]. The number of spindles is proposed to reflect the intrinsic activity of the thalamus [18] as the amplitude and duration would be controlled via the interconnection of cortical and thalamocortical neurons [19]. Finally, spindles characteristics are modulated through developmental changes. Indeed, the amplitude and duration of spindles are known to decrease with the aging process [20–22] and frequency slightly increases [20, 22], whereas sleep becomes shallower and more fragmented [23]. Thus, spindle characteristics (higher amplitude and duration, slower frequency) could be signs of a consolidated sleep spindle, reflecting a lower probability of sleep disturbance. Since it has been shown that INS do show altered, less consolidated sleep [24], their sleep spindles characteristics might differ from those of GS.

INS also display cortical hyperarousal at sleep onset and during sleep compared to GS. In fact, the neurocognitive model of Perlis and collaborators [25], suggesting such hyperarousal in INS, has received much empirical support through spectral analysis. It has been shown that INS exhibit a higher spectral power in the beta frequency bands than control groups at sleep onset, as well as during sleep and wake [26–28], thus reflecting cortical hyperarousal. Besides this higher cortical activation (or hyperarousal), Espie [29] asserted that INS seem to have an inability to inhibit or disengage from active awakening processes. This absent or deficient inhibitory capacity when INS attempt to fall asleep or maintain sleep at night might be associated with a gating problem in information/sensory processing and be the consequence of altered sleep protection mechanisms. As with the neurocognitive model, some studies have provided empirical support for Espie's psychobiological model [30, 31].

Also according to the neurocognitive model, hyperarousal can be observed in the amount or degree of discrepancy between subjective and objective assessments of sleep quality. Indeed, INS overestimate their sleep difficulties [32] and report being awake for long periods of time during the night, even if these observations are not always corroborated by PSG [33, 34]. In addition, INS tend to underestimate their total sleep time (TST) and overestimate the time needed before falling asleep [25]. Perlis and colleagues proposed that hyperarousal contributes to this misperception of sleep quality found in insomnia [25]. To support this relationship between increased cortical activity and sleep misperception, researchers reported a correlation between beta activity and the degree of discrepancy between subjective and objective TST [35]. Other researchers observed a correlation between the ratio delta/beta and misperception of sleep onset latency (SOL [36]). In addition, a subgroup of INS called paradoxical insomnia sufferers (PARA-I) is different from INS suffering from psychophysiological insomnia (PSY-I) since significant dissimilarities are observed between their sleep perception and PSG measures. Those with PARA-I misestimate the quality of their sleep by reporting sleep difficulties while PSG seems normal and similar to GS, compared to PSY-I whose sleep disturbance's perception is supported by PSG. Studies show that PARA-I have a cortical activation pattern even more pronounced than PSY-I [35, 37]. The PARA-I subgroup, which is characterized by a significant underestimation of the quality of their sleep, is also the one having the higher cortical activation. As sleep spindles play a role in the inhibition of sensory processing and thus cortical activation, a greater misperception, that is, a greater discrepancy between objective and subjective sleep parameters, could be linked to less consolidated sleep spindles. Indeed, it is possible that the increased cortical activation in INS, in agreement with the neurocognitive model, may be linked to an alteration of this sleep protection mechanism, thus reducing INS' ability to properly estimate the quality of their sleep.

The objective of the present study is twofold. First it aims at examining sleep consolidation by comparing sleep spindles characteristics between PARA-I and PSY-I and GS. It is predicted that GS will present more consolidated sleep spindles as shown by enhanced number, density, amplitude, and duration and slower frequency. A difference is also foreseen between PARA-I and PSY-I. The latter group, displaying objective sleep difficulties, shall present less consolidated sleep spindles. Secondly, this study also aims at studying the relationship between sleep spindles characteristics and misperception. It is predicted that less consolidated sleep spindles will be associated with an underestimation of sleep quantity, measured by TST.

## 2. Method

### 2.1. Participants

Participants were selected from a former study characterizing evoked potentials in insomnia [38]. Seventeen PARA-I, 24 PSY-I, and 29 GS were randomly pooled from the data bank. The ethical committee of the Université Laval approved this research. All subjects gave an informed consent and received an honorarium for their participation.

#### 2.1.1. Group Classification

The selection of participants was firstly based on their subjective report on two weeks' sleep diaries. Those classified as INS reported sleep difficulties at least 3 nights per week (manifested by sleep onset [SOL] or awakenings of more than 30 min or a total sleep time [TST] of less than 6.5 h). Also, on the* Insomnia Severity Index* (ISI [39]), they had a score of 15 or over and reported at least one moderate daytime consequence due to sleep loss. Finally, their sleep difficulties had to be present for at least 6 months (longer than recommended in the DSM-5) and not be related to a medical, psychological, or another sleep disorder condition or medication intake.

In opposition, good sleepers diaries showed less than three nights with sleep difficulties per week (SOL/awakenings > 30 min or TST < 6.5 h) and a mean TST from 6.5 to 8.5 h per night. On the ISI, GS had a score less than 8. More importantly, they reported no sleep difficulty and no complaint of daytime difficulty related to sleep. An objective SE of less than 85% during the laboratory nights 2 and 3 resulted in an exclusion from the present study.

No rules or guidelines currently set that INS and GS shall be distinguished according to sleep efficiency (SE), which is the result of TST divided by time in bed. Nonetheless, SE has often been used as a reference between good and bad sleepers [4, 15, 40]. What is usually observed is that bad sleepers present a SE lower than 85% while GS show a SE of 85% or more. We have elected to keep this as a reference point so to later be able to verify if participants did have a good or a bad night of sleep, together in consideration with other sleep parameters.

#### 2.1.2. Insomnia Subgroups: Paradoxical and Psychophysiological Insomnia

To ensure that the two subgroups of INS are distinct one from the other, the inclusion criteria for PARA-I and PSY-I match those defined by St-Jean et al. [38]. According to those criteria, PARA-I must present, on two consecutive nights of PSG, (1) a TST over 380 minutes or a SE of 80% and (2) an overestimation of ≥ 60 minutes of their SOL, an underestimation of ≥ 60 minutes of their TST, or ≥ 15% of their SE based on the difference between PSG data and sleep diary data filled in the laboratory on the morning following PSG recordings. INS not meeting these criteria were included in the PSY-I subgroup and those only meeting the second criterion were excluded.

#### 2.1.3. Exclusion Criteria

Exclusion criteria for all participants were as follow: (a) current presence of a medical or neurological disorder that can significantly disrupt sleep; (b) presence of major psychopathology (e.g., anxiety disorders, major depressive disorder); (c) alcohol or drug abuse; (d) evidence of another sleep disorder (e.g., sleep apnea, periodic limb movements during sleep); (e) use of psychotropic or other medications known to alter sleep (e.g., bronchodilators); and (f) use of sleep-promoting agent (e.g., benzodiazepines). Participants using sleep-promoting medication twice a week or less had a 2-week withdrawal period before PSG recordings.

### 2.2. Research Protocol

#### 2.2.1. Procedure

All participants were recruited through local newspapers advertisement. Following a brief telephone screening interview, eligible participants were mailed ethical consent forms and questionnaires to be completed at home, including* Beck Depression Inventory* (BDI [41]);* Beck Anxiety Inventory* (BAI [42]); two weeks of sleep diary to fulfill [39]; and ISI [39]. These questionnaires have all good psychometric properties of validity and reliability (e.g., [41–43]). Following the completion of questionnaires, prospective participants were invited to a clinical interview at the sleep laboratory.* The structured Clinical Interview for DSM-IV Axis I Disorders* [44] and the* Insomnia Diagnostic Interview* [39] were conducted, respectively, by a clinical psychologist and a sleep specialist. Participants who still satisfied the inclusion criteria were invited to spend four nights in the sleep laboratory. They had to avoid any alcohol, drugs, excessive caffeine, and nicotine use before PSG recordings. They arrived at the sleep laboratory around 8:00 pm each night and the sleep technician proceeded to the electrode montage and sleep preparation. Lights-out was initiated after biocalibration. Bedtime was determined according to the sleep diary schedule. Furthermore, time in bed had to exceed 7 h of PSG recordings.

#### 2.2.2. Polysomnographic Recordings

A standard 10–20 system PSG referential montage with linked ears was used, including electroencephalographic (EEG, including C3, C4, O1, and O2), electromyographic (chin), and electrooculographic (left and right: supraorbital ridge of one eye and the infraorbital ridge of the other) recordings. Interelectrode impedance was maintained below 5 kΩ. A Grass Model 15A54 amplifier system (Astro-Med Inc., West Warwick, USA; gain 10,000; bandpass 0.3–100 Hz) was used. On the first night of PSG, respiration and tibialis electromyography were monitored. Participants with sleep apnea (apnea-hypopnea index > 15) and periodic limb movements (myoclonic index with arousal > 15) or diagnosed with another sleep disorder were excluded and referred to an appropriate sleep specialist. PSG signals were digitized at a sampling rate of 512 Hz using commercial software (Harmonie, Stellate System). Qualified technicians visually scored sleep recordings using 20-second epochs (Luna, Stellate System) according to standard criteria [8]. An independent scorer conducted reliability checks (85% interscorer agreement was reached). Night 1 constituted the screening and adaptation night. Night 2 was considered a recovery night. On night 3, auditory sounds (tone pips) were introduced before sleep and after awakening and, on night 4, at sleep onset and throughout the night. The present analyses are derived from the ERPs-free third night in the sleep laboratory as it is the most representative of “usual” sleep of all 4 nights. Objective sleep measures were SOL, wake after sleep onset [WASO], TST, SE (in %), total time (in minutes), and proportion (%) of sleep stages (1, 2, 3, and 4 and rapid eye movement sleep [REM]).

#### 2.2.3. Detection of Sleep Spindles

Sleep spindles detection was conducted on artifact-free N-REM epochs for C3 derivation on night 3 using an algorithm detector [21, 45]. As a recovery effect is often seen on the second night of PSG recordings, and because sleep spindles are relatively stable across nights in individuals, night 3 was considered representative of participants' usual sleep and sleep spindles quality and quantity. Artifacts were automatically detected and rejected [46] and a further visual inspection was made. EEG data was bandpass filtered from 11 to 15 Hz with a linear phase Finite Impulse Response filter (−3 dB at 11.1 and 14.9 Hz). Forward and reverse filtering was performed to obtain zero-phase distortion and double the filter order. The root mean square (RMS) of the filtered signal was then calculated with a 0.25 s time window and a threshold at its 95th percentile [45]. When at least two consecutive RMS time-points exceeded the threshold, reaching duration criterion (0.5 s), a spindle was detected. The density (number/min of stage 2), amplitude (peak-to-peak difference in voltage, in *μ*V), frequency (number of cycles/second, in Hz) and duration (in seconds) were derived for the entire night in stage 2 and SWS (stages 3-4).

#### 2.2.4. Misperception Index

A Total Sleep Time Misperception Index (TST-MI) was calculated using objective (PSG recording) and subjective (sleep diary) data of night 3 using [(TST objective − TST subjective)/TST objective] [47]. A score of 0 is a perfect estimate while a score lower than 0 indicates an overestimation and a score higher than 0 indicates an underestimation of objective data. As sleep spindles are exclusive to sleep, TST was the parameter taken as measure of misperception.

### 2.3. Questionnaires and Interviews

#### 2.3.1. Sleep Diary

The sleep diary [39] is an instrument assessing subjective sleep quality. The respondent daily report sleep wake parameters such as sleep onset latency (SOL, amount of time from initial lights-out to sleep onset), wake after sleep onset (WASO, amount of time awake from sleep onset until the last awakening), early morning awakening (amount of time awake from the last awakening to rising time), frequency of awakenings (total number of awakenings during the night), total wake time (summation of sleep onset latency, wake after sleep onset, and early morning awakening), total sleep time (TST, subtraction of total wake time from the amount of time in bed), and sleep efficiency (SE, ratio of TST divided by time in bed, expressed as a percentage). The sleep diary is completed upon rising for a 2-week baseline period. The mean of each parameter is calculated and thus the diary provides an expanded index of sleep complaints [48].

#### 2.3.2. Insomnia Severity Index (ISI)

The ISI [39] is a valid and reliable self-report instrument measuring perceived insomnia severity [43, 49]. The severity of sleep disturbances, satisfaction relative to sleep, degree of impairment of daytime functioning caused by the sleep disturbances, noticeability of impairment attributed to the sleep problem, and the degree of distress and concern related to the sleep problem are reported throughout the seven items on a 5-point (0 to 4) Likert scale. The total score ranges from 0 to 28 (a higher score reveals more severe insomnia).

#### 2.3.3. Insomnia Diagnostic Interview

The Insomnia Diagnostic Interview [39] is a semistructured interview assessing the presence of insomnia and the potential contributing factors. The nature of the complaint (a), sleep-wake schedule (b), insomnia severity (c), daytime consequences (d), natural history of insomnia (e), environmental factors (f), medication use, (g), sleep hygiene factors (h), presence of other sleep disorders (i), patient's medical history and general health status (j), and a functional analysis of antecedents, consequences, precipitating, and perpetuating factors (k) are parameters surveyed.

### 2.4. Statistical Analyses

Statistical assumptions of parametric tests were verified. In case of violation, nonparametric options were preferred. Kruskal-Wallis *H* tests and chi-square were computed to compare groups on sociodemographic, psychological, and sleep characteristics. Further Kruskal-Wallis tests were used to determine the difference between PARA-I, PSY-I, and GS on sleep spindles characteristics. Thereafter, standard multiple regressions were used to predict TST-MI from sleep spindles characteristics. Significance levels were set at .05. SPSS was used for all analyses (IBM statistics, V23).

## 3. Results

### 3.1. Sociodemographic Variables

The sample comprised 17 PARA-I (11 women, 26–51 years old, mean = 39.36, SD = 9.93), 24 PSY-I (15 women, 28–54 years old, mean = 37.96, SD = 8.65), and 29 GS (19 women, 25–53 years old, mean = 34.83, SD = 9.69). Statistical analysis showed that groups were similar according to gender (*χ* ^2^(2) = 0.021, *p* = 0.990) and education (*H*(2) = 0.991, *p* = 0.609). However, there was a significant difference in regard to age (*H*(2) = 6.049, *p* = 0.049). Furthermore, Kruskal-Wallis *H* test on ISI scores showed a statistically significant difference between groups (*H*(2) = 50.058, *p* < 0.01). Subsequently, pairwise comparisons were performed using Dunn's procedure [50] with a Bonferroni correction for multiple comparisons. Adjusted *p* values are presented. Post hoc tests showed that GS were younger (Mdn = 29.17) than PARA-I (Mdn = 43.24) (*p* = 0.025). In accordance with their condition, both PARA-I (mean rank = 50.25) and PSY-I (mean rank = 49.06) indicated that they experience greater severity of insomnia symptoms than GS (mean rank = 15.50) (*p* < 0.01) as they had significantly higher scores on the ISI. In regard to psychological symptoms, difference between groups were found on depressive and anxious symptoms (BDI, *H*(2) = 14.251, *p* = 0.01; BAI, *H*(2) = 19.452  *p* < 0.01). Both types of INS showed higher BDI scores (PARA-I, mean rank = 36.75; PSY-I, mean rank = 44.07) than GS (mean rank = 24.23) (*p* < 0.01) and higher BAI scores (PARA-I, mean rank = 43.77; PSY-I, mean rank = 40.12) than GS (mean rank = 21.48) (*p* < 0.01), though they remained under the clinical threshold for psychiatric syndrome. There was no difference between PARA-I and PSY-I on the duration of insomnia (*H*(1) = 0.706, *p* = 0.401). Descriptive values can be found in Table 1.

### 3.2. Subjective and Objective Sleep Parameters

Subjective sleep parameters were significantly different between groups for SOL (*H*(2) = 25.497, *p* < 0.001), WASO (*H*(2) = 27.702, *p* < 0.001), TST (*H*(2) = 35.777, *p* < 0.001), and SE (*H*(2) = 48.055, *p* < 0.001). Post hoc tests revealed that both PARA-I (Mdn = 51.47) and PSY-I (Mdn = 40.46) reported a longer time to fall asleep than GS (Mdn = 22.03) as well as being awake longer throughout the night (PARA-I, Mdn = 53.22; PSY-I Mdn = 40.67; GS Mdn = 21.92). PARA-I perceived a shorter TST (Mdn = 10.41) than both GS (Mdn = 47.47) and PSY-I (Mdn = 39.79). Finally, for SE, the three groups reported significant difference (PARA-I, Mdn = 9.65; PSY-I, Mdn = 33.52; GS, Mdn = 52.92). However, regarding objective measures, no significant between-groups difference was found for any measures. Medians and ranges for each sleep parameters (subjective and objective) are depicted in Table 2.

### 3.3. Sleep Spindles Characteristics

Sleep spindles characteristics are presented in Table 3. Analyses showed that spindles duration in stage 2 sleep was significantly different between groups (*H*(2) = 8.769, *p* = 0.012). Post hoc tests with a Bonferroni correction for multiple comparisons revealed that the sleep spindles of PARA-I (mean rank = 26.53) were shorter than GS (mean rank = 41.22; adjusted *p* value = 0.016) but not from PSY-I. Also, the duration was not statistically different between GS and PSY-I. Other characteristics were not significantly different between PARA-I, PSY-I, and GS for either stage 2 or SWS sleep (shown in Table 4).

### 3.4. Misperception and Sleep Spindles Characteristics

Multiple regressions were run to predict TST-MI from sleep spindles characteristics (number, density, duration, frequency, and amplitude) during stage 2 and SWS and from objective sleep measures (time and percentage spent in each stage, SOL, WASO, TST, and SE). Foremost, 32 of 71 participants overestimated (8 PSY-I and 25 GS) and 38 of 71 underestimated (17 PARA-I, 16 PSY-I and 5 GS) their TST. The multiple regression models showed that no variable added to the models significantly predicted the overestimation (*F*(20,11) = 1.425, *p* = 0.277) or underestimation (*F*(21,13) = 2.320, *p* = 0.061) of TST. Regression coefficients and standard errors can be found in Tables 5 and 6.

## 4. Discussion

The first objective of this study was to establish if significant differences in sleep spindles characteristics (number, density, duration, frequency, and amplitude) existed between good sleepers and insomnia individuals in order to further investigate the protective factors of sleep in insomnia. The second aim was to study the relationship between sleep spindles and misperception, that is, if lower consolidated sleep spindles would be associated with an underestimation of sleep quality.

### 4.1. Sleep Parameters

Insomnia is a condition marked by subjective sleep difficulties. Mainly, INS report longer SOL and WASO and shorter TST on questionnaires such as sleep diaries [4, 40, 51]. In the present study, we further divided INS into two groups: PARA-I and PSY-I. Our two groups also reported taking more time to fall sleep, being awake longer during the night as well as having a lower sleep efficiency than GS. Furthermore, PARA-I perceived sleeping less than GS. Contrary to earlier reports [4–6], PSY-I presented similar PSG to good sleepers. These surprising results can nonetheless be explained by the variability in the sleep patterns of INS. Indeed, chronic insomnia is characterized by great internight variability, with alternations between good and bad nights in a typical week [52]. This variability in sleep patterns can also be identified during laboratory nights, both for INS and GS. Indeed, according to St-Jean and Bastien [53], on three consecutive nights in the laboratory, around half of the INS reported having at least one good night and conversely about half of GS at least one bad night. More specifically, on night 3, slightly over than one-third of INS reported having a good night while a quarter of GS reported having a bad night. As such, sleep representativeness in each group is reduced. Since the data of our study comes only from the third night, it is possible that this night was not as representative of sleep difficulties as usually reported by INS or good sleepers' adequate sleep quality.

### 4.2. Sleep Protection in Insomnia

The results indicate that the characteristics of sleep spindles are similar between PARA-I, PSY-I, and GS except for spindle duration in stage 2 sleep that is significantly shorter for PARA-I than for GS but is not different between PSY-I and GS. The number and density of sleep spindles as a protective mechanism of sleep seem intact in PSY-I and PARA-I. These observations corroborate previous results obtained by Bastien and colleagues in PSY-I [15]. Furthermore, in this present study, the amplitude and frequency were not distinctive features separating groups. The amplitude of the sleep spindle is still understudied in the literature while reports considering the frequency of spindles are usually limited to general cognitive ability's hypothesis differentiating between fast (~13-14 Hz) and slow spindles (~11-12 HZ), making it difficult to conciliate our results with existent data.

Results revealed that GS showed longer duration of sleep spindles than PARA-I. The literature has shown that a spindle is created from the interaction of inhibitory neurons of the nucleus reticularis thalami (nRT), excitatory thalamocortical neurons (TC), and finally, progressively, cortical neurons [54]. As mentioned earlier, a negative correlation between the probability of nRT neurons to be involved in the first cycle of sleep spindle and the duration of the spindle was observed in cats [16]. In fact, a spindle of long duration, in comparison to spindles presenting a short one, recruits less neurons in the first cycle but shows a progressive increase, reaching a plateau, than a progressive decrease. In that sense, the protection value could be in the continuity of recruitment compared to its strength. As such, GS would then benefit from a longer sustained protection than PARA-I. This result could also be evaluated in relation to density. As the presence of the sleep spindle seems to be identical in terms of spindle per minute, a difference in length of these phasic events could influence the sleep maintenance. It is also important to note that, in our sample, GS were significantly younger in age than PARA-I. Age has been related to spindles length as younger individuals do show longer spindle duration and it is possible that the difference in sleep spindles duration is driven by this age difference.

This result, however, contradicts our hypothesis, which postulated less consolidated sleep spindles in PSY-I to maintain sleep. In our sample, the fact that PSY-I showed no objective sleep differences compared with PARA-I or GS may be the reflection of a good consolidated night in the laboratory. Furthermore, sleep consolidation in PARA-I could also be studied by sleep microstructure, which is sometimes more attuned with EEG arousals. For example, the microstructure could be studied through microarousals, being defined as a brief increase in EEG frequency that could include theta, alpha, and/or frequencies greater than 16 Hz, excluding spindles [55] or by EEG cycling alternating patterns (CAPs) which are fluctuations in arousal translating in an arousal phase (A1 to A3) followed by a quiet sleep phase (B [56]). Feige et al. found that INS did have more microarousals in both REM and N-REM sleep ([57], corrigendum [58]). Furthermore, Parrino et al. [59] reported a significant higher total CAP rate (58% versus 35%), particularly in A2 subtypes (31% versus 24%), in PARA-I compared to controls, reflecting an instable sleep pattern in those individuals. Thus, the difference that we found between GS and PARA-I instead of PSY-I may be the reflection of their sleep instability measured by these two signs of arousal.

### 4.3. Sleep Misperception

Cortical hyperarousal has been shown to be related to the misperception of sleep quality in PARA-I, especially through an increase in sensory and information processing when falling asleep and during sleep-wake transitions, making the distinction between periods of sleep and wakefulness less obvious [60]. In an empirical study, sleep spindles appearing concomitantly with sounds (e.g., tone pips) have been shown to protect sleep [13]. An underlying hypothesis that could be postulated is that the presence of spindles also influences an individual's perception of sleep by stopping the processing of information. However, according to our results, the sleep spindles characteristics do not seem to have a predictive value on the perception of being asleep. Yet, a topographical approach to sleep misperception could be more suitable as some evidence suggests that PARA-I exhibit a disturbance in neural synchronization on anterior derivations, reflected by more beta and less delta and sigma power [61]. Because our study focused solely on sleep spindles from C3, we did not study distinctive patterns in different scalp areas, which may have contributed differently to sleep perception. Indeed, some differences in spindles characteristics, according to each derivation, were previously observed by other researchers [21].

The present study has some limits. First, the use of algorithms to detect sleep spindles is generally associated with false positive [62]. This can lead to the detection of various sleep EEG elements other than sleep spindles and thus a reduced specificity in the representation of the sleep protection mechanisms. However, automatic detectors are objective tools, mainly for different characteristics' analysis (amplitude, duration, and frequency). In addition, algorithms are usually more precise for detecting spindles in SWS. Secondly, as stated earlier, differences at distinct derivations in sleep spindles characteristics remain to be circumscribed.

## 5. Conclusion

In summary, in the present study, the evidence regarding sleep spindle as a sleep protection mechanism remains unclear. However, sleep spindles duration in stage 2 differed between PARA-I and GS, as the length of spindles is shorter for this insomnia subgroup. The implications of this result are uncertain, the literature being scarce regarding the different functions of the characteristics of the sleep spindle.

Future studies could investigate the occurrence of sleep spindles in insomnia sufferers following a night of sleep deprivation so to provide additional information about their role in sleep protection mechanisms and their homeostatic modulation in these individuals. Indeed, some researchers have shown a reduction in the density of sleep spindles after partial or total sleep deprivation [63–65]. This decrease is explained by an increase in slow-waves, thus supporting a reciprocal relationship between these EEG's elements. It would also be interesting to study sleep spindles during sleep transition (between stages and with respect to microarousals). Actually, Wauquier et al. [66] found a reduced number of *K*-complexes in the 5 min ascending and descending phases of sleep 2 stages. It is possible that the stage-shift period is particularly susceptible to deficient consolidation and sleep protection in insomnia individuals. Furthermore, since the *K*-complex has also been associated with sleep protection [67], studying spindles characteristics according to the presence of a *K*-complex (before or after) could shed some light on the respective behaviors of these graphoelements during sleep in insomnia sufferers.

## Figures and Tables

**Table 1:** Medians (ranges) of sociodemographic data of good sleepers (GS) and paradoxical (PARA-I) and psychophysiological (PSY-I) insomnia sufferers.

	GS = 29	PARA-I = 17	PSY-I = 24
Age (years)	31.00 (25.00–53.00)^b^	42.00 (26.00–51.00)^b^	37.00 (28.00–54.00)
Gender (women/men)	19/10	11/6	15/9
Education (years)	16.00 (6.00–21.00)	16.00 (6.00–17.00)	16.00 (6.00–25.00)
Insomnia length (months)	—	96.00 (12.00–360.00)	96.00 (3.00–360.00)
*Questionnaires*			
ISI	1.00 (0.00–7.00)^b,c^	16.00 (11.00–22.00)^b^	17.00 (1.00–22.00)^c^
BDI	2.00 (0.00–12.00)^b,c^	5.00 (1.00–10.00)^b^	7.00 (0.00–20.00)^c^
BAI	2.00 (0.00–11.00)^b,c^	6.00 (2.00–20.00)^b^	5.00 (0.00–13.00)^c^

*Note.* ISI = Insomnia Severity Index. BDI = Beck Depression Inventory. BAI = Beck Anxiety Inventory. b: significant difference between the PARA-I and GS. c: significant difference between the PSY-I and GS.

**Table 2:** Medians (ranges) of laboratory night 3 objective and subjective sleep parameters of good sleepers (GS) and paradoxical (PARA-I) and psychophysiological (PSY-I) insomnia sufferers.

	GS	PARA-I	PSY-I
*Subjective*			
SOL (min)	8.00 (0.00–25.00)^b,c^	30.00 (0.00–120.00)^a,b^	15.00 (8.00–60.00)^a,c^
WASO (min)	3.00 (0.00–45.00)^b,c^	60.00 (0.00–240.00)^a,b^	17.50 (0.00–120.00)^a,c^
TST (min)	450.00 (350.00–516.00)^b^	327.50 (0.00–390.00)^a,b^	420.00 (332.00–491.00)^a^
SE (%)	95.83 (82.97–100.00)^b,c^	69.38 (11.76–81.30)^a,b^	88.20 (69.40–95.83)^a,c^
*Objective*			
SOL (min)	6.00 (1.00–31.33)	5.67 (2.00–32.67)	6.67 (1.33–30.33)
WASO (min)	16.33 (3.67–64.00)	29.50 (5.00–44.67)	22.34 (1.00–136.67)
TST (min)	424.00 (345.67–491.00)	419.00 (371.33–458.33)	424.67 (335.00–507.00)
SE (%)	94.00 (85.00–98.00)	90.50 (88.00–96.00)	92.50 (72.00–99.00)
*Stage 1*			
Total time (min)	8.00 (1.00–32.33)	6.34 (1.33–22.67)	10.67 (1.67–35.33)
Proportion (%)	1.82 (0.21–8.44)	1.52 (0.32–5.67)	2.86 (0.45–7.46)
*Stage 2*			
Total time (min)	257.67 (170.00–350.00)	248.67 (175.00–324.67)	276.67 (148.33–360.00)
Proportion (%)	62.69 (40.28–71.28)	61.24 (41.63–72.26)	64.13 (33.36–79.76)
*SWS*			
Total time (min)	21.67 (0.84–62.67)	20.50 (4.50–73.50)	12.92 (0.00–97.00)
Proportion (%)	4.87 (0.23–14.51)	4.78 (0.99–17.49)	2.93 (0.00–21.82)
*REM *			
Total time (min)	114.67 (60.00–169.33)	103.84 (63.67–139.67)	103.34 (67.33–164.67)
Proportion (%)	26.85 (17.36–37.64)	25.04 (17.15–32.67)	25.66 (14.92–37.97)

*Note.* SOL: sleep onset latency. WASO: wake after sleep onset. TST: total sleep time. SE: sleep efficiency. SWS: slow wave sleep. REM: rapid eye movement. a: significant difference between the PARA-I and PSY-I. b: significant difference between the PARA-I and GS. c: significant difference between the PSY-I and GS.

**Table 3:** Characteristics of sleep spindles for good sleepers (GS) and paradoxical (PARA-I) and psychophysiological (PSY-I) insomnia sufferers.

	GS	PARA-I	PSY-I
*Stage 2*			
Number	829.50 (508.00–1126.00)	812.00 (597.00–1151.00)	871.00 (596.00–1196.00)
Density (spindles/min)	3.30 (2.40–4.06)	3.33 (2.49–3.81)	3.51 (2.70–4.21)
Duration (sec)	0.70 (0.60–0.70)	0.60 (0.60–0.70)	0.70 (0.60–0.70)
Frequency (Hz)	13.25 (12.50–13.60)	13.10 (12.70–13.70)	13.10 (12.80–13.40)
Amplitude (*μ*v)	28.40 (16.30–61.40)	31.50 (21.10–42.70)	29.15 (19.10–45.00)
*SWS*			
Number	69.50 (1.00–258.00)	58.00 (11.00–339.00)	54.50 (6.00–612.00)
Density (spindles/min)	1.81 (0.20–4.12)	2.14 (0.95–3.39)	1.65 (0.59–3.20)
Duration (sec)	0.55 (0.50–0.75)	0.55 (0.50–0.70)	0.55 (0.50–0.70)
Frequency (Hz)	13.18 (12.00–14.00)	13.00 (12.40–13.85)	13.10 (12.15–13.60)
Amplitude (*μ*v)	27.17 (14.20–63.10)	28.65 (21.50–40.50)	27.40 (17.50–44.05)

*Note.* SWS: slow wave sleep.

**Table 4:** Difference between good sleepers, paradoxical and psychophysiological insomnia sufferers according to sleep spindles characteristics on night 3.

Spindles characteristics	Stage 2		SWS	
	*H *value (Kruskal-Wallis)	*p*	* H* value (Kruskal-Wallis)	*p*
Number	1.613	0.446	1.496	0.473
Density (spindles/min)	1.329	0.525	1.832	0.400
Duration (sec)	8.769	0.012^*∗*^	1.200	0.549
Frequency (Hz)	2.890	0.236	0.814	0.666
Amplitude (*μ*v)	1.076	0.584	1.093	0.579

*Note.∗*: significant difference between the groups. SWS: slow wave sleep.

**Table 5:** Summary of multiple regression analysis for overestimation (Misperception Index < 0).

Variable	*B*	SE_*B*_	Beta
Intercept	−0.977	1.432	
*Stage 2 sleep spindles*			
Number	0.000	0.000	−0.804
Density	0.059	0.078	0.581
Duration	0.135	0.238	0.147
Frequency	−0.016	0.057	−0.107
Amplitude	0.002	0.002	0.391
*SWS sleep spindles*			
Number	−0.001	0.001	−1.892
Density	0.076	0.027	1.482
Duration	0.071	0.184	0.112
Frequency	0.004	0.029	0.054
Amplitude	−0.002	0.002	−0.634
*Objective sleep measures*			
SOL (min)	0.002	0.002	0.314
WASO (min)	0.001	0.002	0.329
TST (min)	0.001	0.002	0.718
SE (%)	0.006	0.012	0.580
Stage 1 (min)	−0.042	0.025	−7.448
Stage 1 (%)	0.169	0.100	7.587
Stage 2 (min)	—	—	—
Stage 2 (%)	—	—	—
Stage SWS (min)	−0.015	0.011	−5.435
Stage SWS (%)	0.071	0.047	6.243
REM (min)	−0.039	0.200	−5.111
REM (%)	0.008	0.005	5.436

*Note.∗* = *p* < 0.05; *∗∗* = *p* < 0.01.  *B*: unstandardized regression coefficient; SE_*B*_: standard error of the coefficient; beta: standardized coefficient. SOL: sleep onset latency. WASO: wake after sleep onset. TST: total sleep time. SE: sleep efficiency. SWS: slow wave sleep. REM: rapid eye movement.

**Table 6:** Summary of multiple regression analysis for underestimation (Misperception Index > 0).

Variable	*B*	SE_*B*_	Beta
Intercept	2.241	16.820	
*Stage 2 sleep spindles*			
Number	−0.002	0.001	− 1.721
Density	0.202	0.374	0.316
Duration	−1.077	0.691	−0.239
Frequency	−0.208	0.253	−0.226
Amplitude	0.062	0.018	1.789
*SWS sleep spindles*			
Number	−0.002	0.001	−1.051
Density	0.167	0.087	0.525
Duration	−1.104	0.929	−0.227
Frequency	−0.041	0.182	−0.067
Amplitude	−0.046	0.016	−0.227
*Objective sleep measures*			
SOL (min)	−0.005	0.017	−0.160
WASO (min)	−0.005	0.015	−0.603
TST (min)	−0.007	0.014	−1.053
SE (%)	−0.045	0.085	−0.917
Stage 1 (min)	−0.099	0.063	−3.495
Stage 1 (%)	0.505	0.337	4.185
Stage 2 (min)	0.005	0.019	1.096
Stage 2 (%)	0.096	0.093	3.987
Stage SWS (min)	—	—	—
Stage SWS (%)	0.212	0.248	4.730
REM (min)	0.043	0.024	4.733
REM (%)	−0.117	0.139	−2.608

*Note.∗* = *p* < 0.05; *∗∗* = *p* < 0.01.  *B*: unstandardized regression coefficient; SE_*B*_: standard error of the coefficient; beta: standardized coefficient. SOL: sleep onset latency. WASO: wake after sleep onset. TST: total sleep time. SE: sleep efficiency. SWS: slow wave sleep. REM: rapid eye movement.

## Glossary

BAI: Beck Anxiety Inventory
BDI: Beck Depression Inventory
EEG: Electroencephalogram
GS: Good sleeper
INS: Insomnia sufferer
ISI: Insomnia Severity Index
N-REM: Nonrapid eye movement
PARA-I: Paradoxical insomnia sufferers
PSG: Polysomnography
PSY-I: Psychophysiological insomnia sufferers
SE: Sleep efficiency
SOL: Sleep onset latency
SWS: Slow wave sleep
TST: Total sleep time
TST-MI: Total Sleep Time Misperception Index
WASO: Wake after sleep onset.
